# *Anopheles sundaicus*  complex and the presence of *Anopheles epiroticus* in Indonesia

**DOI:** 10.1371/journal.pntd.0008385

**Published:** 2020-07-02

**Authors:** Din Syafruddin, Yulia E. Lestari, Dendi H. Permana, Puji B. S. Asih, Brandyce St. Laurent, Siti Zubaidah, Ismail E. Rozi, Sully Kosasih, Shinta, Supratman Sukowati, Lukman Hakim, Edhi Haryanto, Wibowo Mangunwardoyo, Michael J. Bangs, Neil F. Lobo

**Affiliations:** 1 Eijkman Institute for Molecular Biology, Jakarta, Indonesia; 2 Department of Parasitology, Faculty of Medicine, Hasanuddin University, Makassar, Indonesia; 3 Eck Institute for Global Health, University of Notre Dame, IN, United States of America; 4 Health Ecology Research & Development Centre, National Institute of Health, Research and Development, Ministry of Health, Jakarta, Indonesia; 5 Division of Vector Borne Disease, Ministry of Health, Jakarta, Indonesia; 6 Department of Biology, Faculty of Mathematics and Science, Universitas Indonesia, Depok, Indonesia; 7 PT Freeport Indonesia, International SOS, Freeport Medical Services, Kuala Kencana, Papua, Indonesia; 8 Department of Entomology, Faculty of Agriculture, Kasetsart University, Bangkok, Thailand; Centers for Disease Control and Prevention, UNITED STATES

## Abstract

*Anopheles sundaicus* s.l. is an important malaria vector primarily found in coastal landscapes of western and central Indonesia. The species complex has a wide geographical distribution in South and Southeast Asia and exhibits ecological and behavioural variability over its range. Studies on understanding the distribution of different members in the complex and their bionomics related to malaria transmission might be important guiding more effective vector intervention strategies. Female *An*. *sundaicus* s.l. were collected from seven provinces, 12 locations in Indonesia representing Sumatra: North Sumatra, Bangka-Belitung, South Lampung, and Bengkulu; in Java: West Java; and the Lesser Sunda Islands: West Nusa Tenggara and East Nusa Tenggara provinces. Sequencing of ribosomal DNA ITS2 gene fragments and two mitochondrial DNA gene markers, *COI* and *cytb*, enabled molecular identification of morphologically indistinguishable members of the complex. Findings allowed inference on the distribution of the *An*. *sundaicus* s.l. present in Indonesia and further illustrate the phylogenetic relationships of *An*. *epiroticus* within the complex. A total of 370 *An*. *sundaicus* s.l specimens were analysed for the ITS2 fragment. The ITS2 sequence alignment revealed two consistent species-specific point mutations, a T>C transition at base 479 and a G>T transversion at base 538 that differentiated five haplotypes: TG, CG, TT, CT, and TY. The TG haplotype matched published *An*. *epiroticus–*indicative sequences from Thailand, Vietnam and peninsular Malaysia. The previously described insertion event (base 603) was observed in all identified specimens. Analysis of the *COI* and *cytb* genes revealed no consistent nucleotide variations that could definitively distinguish *An*. *epiroticus* from other members in the Sundaicus Complex. The findings indicate and support the existence of *An*. *epiroticus* in North Sumatra and Bangka-Belitung archipelago. Further studies are recommended to determine the full distributional extent of the Sundaicu*s* complex in Indonesia and investigate the role of these species in malaria transmission.

## Author summary

In Indonesia, more than 80 species of *Anopheles* have been documented, of which approximately 24 species are regarded as malaria vectors. Many of these mosquitoes are members of species complexes, being morphologically indistinguishable from other member sibling species. The *Anopheles sundaicus* complex (s.l.) has a wide geographical distribution in South and Southeast Asia, and exhibits ecological and behavioural variability over its range. *Anopheles sundaicus* s.l. is an important malaria vector present primarily in coastal areas of western and central Indonesia. Cytologic, isozyme, and molecular methods have been used to distinguish members of the complex in Indonesia, with several species being reported in different localities. This study aims to explore the distribution of the *An*. *sundaicus* complex in Indonesia using molecular methods. Female *Anopheles sundaicus* s.l. were collected from 12 localities within seven provinces in western to south-central Indonesia. The findings indicate the presence of *An*. *epiroticus* in North Sumatra and Bangka-Belitung archipelago off the eastern coast of central Sumatra. Further studies are required to determine the full distributional extent of the *An*. *sundaicus* complex in Indonesia and investigate the role of each species in malaria transmission.

## Introduction

The genus *Anopheles* contains over 480 formally described species worldwide [1] with more awaiting description. Though over 100 *Anopheles* species have the capacity to transmit human malaria parasites, only a handful of species are currently regarded as ‘primary’ vector species, some of which are members of species complexes [2–4]. In Indonesia, more than 80 species of *Anopheles* are known present, with approximately 24 confirmed as malaria vectors [5]. Many of these mosquitoes are members of sibling species complexes, genetically closely related taxa morphologically indistinguishable from one another. Behavioural differences between members of a species complex may dramatically influence their respective capacity to transmit pathogens [6–7]. For example, the *Anopheles farauti* complex present in Papua New Guinea has at least three members as important malaria vectors, while four others are either secondary or non-vectors [8]. Possible behavioural and biological differences between species that contribute to pathogen transmission may determine whether their specific identification is useful for enhancing malaria control programs.

*Anopheles* (*Cellia*) *sundaicus* (Rodenwaldt) is an important malaria vector throughout its range in Indonesia [4]. This species has a wide distribution throughout Indonesia (Sumatra to the Maluku islands), the only major exclusion is the western half of New Guinea Island. It is present primarily in coastal zone habitats and more limited in interior areas and foothills [9–11]. *Anopheles sundaicus* is typically anthropophilic; however, host preference and biting behaviour may vary by location and host availability [4]. Immature stages of *An*. *sundaicus* are typically associated with coastal, sunlit, brackish water habitats, and often with floating algal mats [4]. Salinity of coastal habitats typically ranges between 1.2 to 1.8% (seawater is ~3.5%). For inland areas at higher elevations (up to 1000 m), freshwater habitats are used [11–15].

The *An*. *sundaicus* complex is in the subgenus *Cellia*, Pyretophorus Series [1]. Members in the complex can be easily identified morphologically, whereas definitive Species identifications are based on cytogenetic (ovarian polytene chromosomes), specific enzymatic (isozymes) presence, and molecular (DNA) markers. The initial discovery of sibling species involved three cytological forms from populations in Thailand, Sumatra and Java, provisionally designated cytotypes (forms) A, B and C [16] and supported by isozyme evidence [17]. Cytotype D was later identified in the Andaman and Nicobar Islands (Indian Ocean) [18, 19]. Form A is the most widespread of the three cytotypes, occurring primarily along coastal, brackish water ecologies in Thailand and Indonesia (Sumatra and Java). The molecular method uses DNA markers—the nuclear ribosomal DNA (rDNA) Internal Transcribed Spacer-2 (ITS2), and the mitochondrial DNA (mtDNA) Cytochrome Oxidase I (*COI*) and Cytochrome b (*Cytb*) genes. Four members of the *An*. *sundaicus* complex have been identified molecularly: *An*. *sundaicus* s.s., *An*. *epiroticus* Linton & Harbach, *An*. *sundaicus* species D, and *An*. *sundaicus* species E [20].

The use of chromosome and DNA to identify sibling species has resulted in discrepant conclusions between the two methods. Cytologic evidence indicates that Form A (= *An*. *epiroticus*) should be present in Sumatra and Java [16, 17, 21]. However, this is contradictory with more current findings based on DNA evidence indicating that *An*. *epiroticus* appears confined to mainland Southeast Asia and not present in Indonesia or eastern Malaysia (Borneo) [20, 22, 23]. Form B has been found in sympatry with Form A at inland freshwater sites near Purworejo (south-central Java), and South Tapanuli (northern Sumatra). Form C appears confined to a coastal locality in the Asahan area in north-eastern Sumatra and also sympatric with forms A and B [16, 17].

*Anopheles sundaicus* s.s. [24] from Sarawak (Malaysian Borneo) [14] and *An*. *epiroticus* from mainland Southeast Asia [21] are allopatric. Because sibling species are isomorphic, lacking sufficient morphological characters for definitive species determination, DNA markers using fixed differences in the rDNA ITS2 fragment and the mtDNA *COI* gene are used to separate the two species. Allopatric *An*. *sundaicus* species D (Indian Ocean) is based on chromosomal forms and ITS2 sequences [18, 19]. Until very recently, *An*. *sundaicus* species E in Indonesia (e.g., Sumatra, Java and Sumba islands) was considered allopatric with the other Sundaicus Complex members with separation based on *COI* sequences using a multiplex allele-specific polymerase chain reaction [20, 22, 25–27]. So far, the relationships of cytotypes B and C to other members in the complex remain unclear [22, 25].

The present study explores the distribution, phylogenetic relationships, and population structure of the *An*. *sundaicus* complex in Indonesia. Morphological characters were compared with clades identified from DNA markers—genes encoding mtDNA *COI* and cytochrome-*b* (*cytb*), and the rDNA ITS2 region. The *COI* sub-unit gene has been widely used in anopheline systematics and population structure studies [25], while the ITS2 region shows low co-evolutionary intra-specific and high inter-specific variation [28], and serves a valuable marker for the molecular characterization of anopheline species [29, 30].

## Methods

### Ethics statement

This study was approved by the Ethics Committee of Research in Health, Medical Faculty of Hasanuddin University, Makassar, Indonesia (No.0868/H4.8.4.5.31/PP36-KOMETIK/2011) and endorsed by the Eijkman Institute Research Ethics Committee, Jakarta, Indonesia (No. 51; 29 December, 2011). Both committees include a certified veterinarian to oversee and ensure the well-being of animals (animal-baited trapping) used in this study.

### Mosquitoes

**Fig 1:**
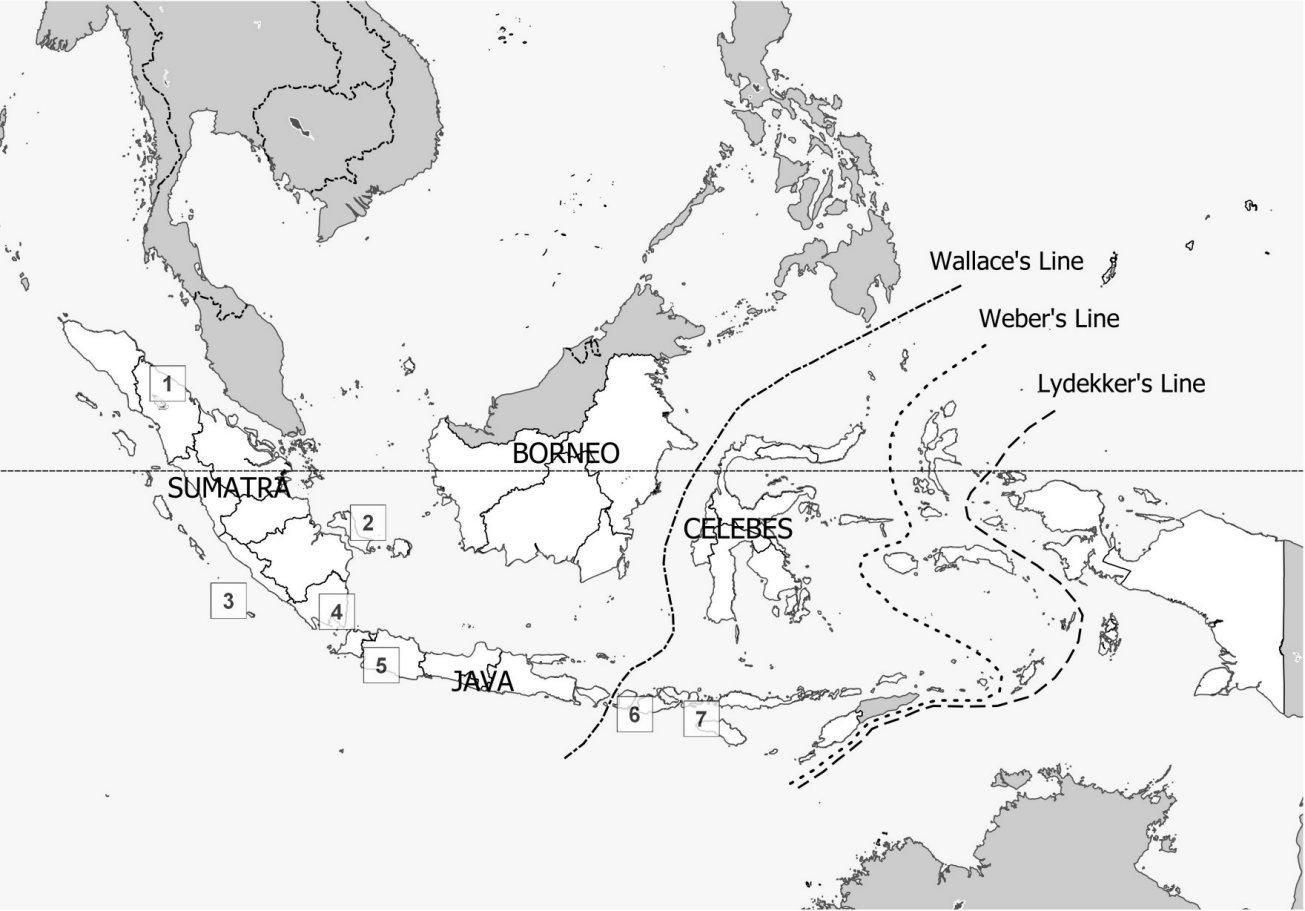
The seven collection areas (provinces), comprising 12 collection sites for *An*. *sundaicus* s.l. in Indonesia (1) North Sumatra, (2) Bangka-Belitung, (3) Bengkulu (Enggano Is.), (4) South Lampung, (5) West Java, (6) West Nusa Tenggara, and (7) East Nusa Tenggara. The dashed lines are biogeographical transition zones the separate Asia and Australasia. The transitional zone between the Wallace’s and Weber’s lines is termed ‘Wallacea’. Plants and animals related to Asian species are found to the north-west. Australasian species are found mainly to the south-east, with a small inter-mix of Asian species as seen with *An*. *sundaicus*. This species complex is not found east of Weber’s or Lydekker’s lines. Map from Natural Earth. https://www.naturalearthdata.com/.

Female *Anopheles* mosquitoes were collected as part of independent studies on malaria and mosquito bionomics in Indonesia between 2007 and 2015. Geo-coordinates of collection locations are provided in Table 1. Mosquitoes were collected using human-landing catches (HLC), animal-baited traps, and larval collections, from seven provinces: North Sumatra, Bangka-Belitung islands, South Lampung, Enggano Island (Bengkulu), West Java, West Nusa Tenggara, and East Nusa Tenggara (Fig 1), representing 12 specific locations (Table 1).

**Table 1:** Inter-specific variable bases of the ITS2 region that distinguish other *Anopheles sundaicus* members from *Anopheles epiroticus*.

Study Site		Latitude/Longitude	Variable bases at nucleotide 479 and 538 –ITS2 sequence					N
			*An*. *epiroticus*	*An*. *sundaicus* s.l.				
Province	Village		T*G^+^	CG	TT	CT	YK	
North Sumatra	Barbaran, Sabajior	0.852777 N: 99.526828 E0.815799 N: 99.543322 E	14	7	-	-	-	21
Bangka-Belitung	Keciput	2.576194 S: 107.820301 E	138	1	2	1	9	151
Bengkulu	Banjar Sari, Apoho, Kahyapu	5.291862 S: 102.163561; E5.348357 S: 102.272781; E5.420082 S: 102.370094 E	-	10	-	-	-	10
South Lampung	Canti, Sukajaya Lempasing	5.795917 S: 105.586181; E5.500211 S: 105.251934 E	-	68	-	33	-	101
West Java	Penanjung	7.676395 S: 108.648675 E	-	1	-	1	-	2
West Nusa Tenggara	Selengen	8.235628 S: 116.302685 E	-	3	-	5	-	8
East Nusa Tenggara	Gaura, Wainyapu	9.731046 S: 119.265754; E9.640085 S: 119.015318 E	-	76	-	1	-	77
Total			152	156	2	41	9	370

*Anopheles epiroticus*, TG [21]

(* = Base 479

^+^ = Base 538 of ITS2 region; Y = C/T)

### Human landing catch

At the beginning of a study in each site, local people were asked their willingness to participate in HLCs. After obtaining informed consent, healthy adult male and female volunteers, were trained to collect mosquitoes that landed on their exposed legs using a mouth aspirator and place them in holding cups. Each collection night, HLCs were conducted both indoors and outdoors from 18.00–06.00 h with a 10-minute rest period every hour. The number of collection nights varied by study. After study participation, HLC volunteers were contacted for one month for tracking and treating any possible mosquito-borne infections the result of their participation.

### Animal-baited tent trap

Animal-baited tent traps utilized local livestock. Traps were located near livestock stables with either a cow or a goat leashed loosely to a stake in the center of the tent enclosure. Evening-active mosquitoes found resting on the inner surface of the tenting material were collected using a mouth aspirator once each hour, from 18.00 to 06.00.

### Sample processing

Female mosquitoes were initially identified using morphological keys by field staff [31, 32], and preserved individually in 1.5 ml snap-cap plastic tubes over silica gel separated with a cotton plug preventing direct contact between desiccant and specimen [14]. Those identified as *An*. *sundaicus* were separated, and preserved for later detailed morphological species confirmation and molecular analysis.

### Molecular procedures

DNA was extracted from individual adult mosquitoes using Chelex-100 (BioRad Laboratories, Hercules, CA, USA) with slight modifications to the protocol [33]. Briefly, mosquitoes were ground with clean Teflon pestles in 50 μl blocking buffer (BB), containing 5.0 g Casein; 0.01 g/L Phenol Red; 900 ml phosphate buffered saline (PBS), pH 7.4; 100 ml of 0.1 N NaOH; with additional IGEPAL (5 μl IGEPAL: 1 ml BB). Afterwards, the pestles were rinsed with additional 200 μl of blocking buffer. Mosquito DNA from the homogenate was extracted using the Chelex-100 ion exchanger. The 50 μl homogenate was added to 50 μl 20% Chelex-100 in distilled water (pH ≥ 10.5). The DNA was extracted by boiling at 100°C for 10 min. The DNA was either used immediately for polymerase chain reaction (PCR) or stored at -20ºC for later analysis.

### PCR amplification and DNA sequencing

Amplification of the *COI*, *cytb*, and ITS2 genomic regions was performed using each respective set of gene primers [20, 25, 28]. The PCR products were purified using clean-up systems (PROMEGA Corporation, Madison, WI, USA) and Exonuclease I—shrimp alkaline phosphatase (USB, Affymetrix, Cleveland, OH, USA). The purified amplicons were sequenced using an ABI Prism Dye BigDye Terminator Cycle Sequencing Ready Kit (Applied Biosystem, Foster City, CA, USA) in a fluorescent DNA capillary electrophoresis sequencer (ABI 3130×l) at the Eijkman Institute (Jakarta, Indonesia), and repeated at the University of Notre Dame (Indiana, USA).

### Analysis

Phylogenetic analyses were performed by aligning DNA sequences using ClustalW, an alignment editor (Biological Sequence Alignment Editor, BioEdit, ver 7.0.9 Ibis Biosciences, Carlsbad, CA, USA), BLAST (GenBank, NCBI), and manual examination. Multiple alignments were made using Molecular Evolutionary Genetics Analysis (MEGA) Software Version 6.0. Phylogenetic trees were constructed using 1000 bootstrap re-sampling repetitions. Evolutionary distances were computed using the Maximum Composite Likelihood method and are in the units of the number of base substitutions per site. The percentage of replicate trees and associated taxa that clustered together in the bootstrap test was also calculated [34]. The analysis utilized 17 ITS2 and 20 *COI* nucleotide sequences. All ambiguous positions were removed from each sequence pair resulting in 344 positions in the final dataset. Evolutionary analyses used MEGA6 [35, 36]. The *An*. *subpictus* ITS2 (GenBank accession number KX622063), and COI (GenBank accession number KJ461780) gene sequences were used as outgroups in the molecular analyses.

## Results

Approximately 90% of the initially field-identified *An*. *sundaicus* s.l. specimens were confirmed as *An*. *sundaicus* s.l. using follow-up expert morphological examination and molecular-based methods. DNA sequences were generated from 370 *An*. *sundaicus* specimens collected from northern Sumatra to Sumba Island located in East Nusa Tenggara Province, geographically spanning most of the known range of this species complex in Indonesia (Fig 1). Target sequences included the ITS2 (n = 370), partial *COI* (n = 107), and *ctyb* (n = 107) genes.

### PCR and sequencing ITS2 fragments

**Fig 2:**
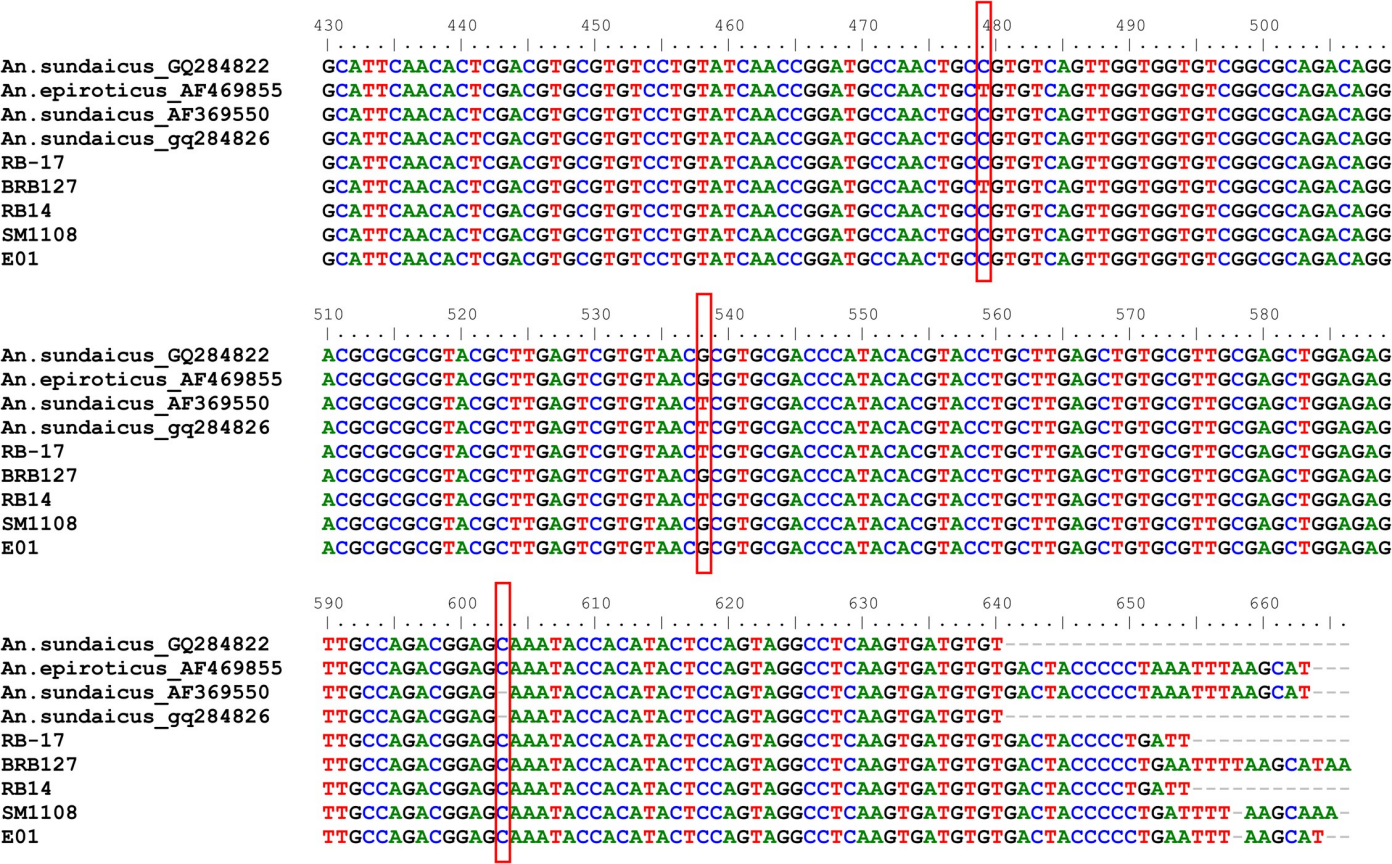
Alignment of rDNA ITS2 sequences from *An*. *sundaicus* s.l. (GenBank Acc. No. GQ284822, AF369550, GQ284826) and *An*. *epiroticus* (GenBank Acc. No. AF469855). Geographic origins of specimens indicated as RB-17 = Lampung, BRB-127 = North Sumatra, RB-14 = Lampung, SM1-108 = Sumba, East Nusa Tenggara, and E-01: Enggano, Bengkulu.

All 370 *An*. *sundaicus* specimens successfully yielded a 586 base pair (bp) ITS2 amplicon. The rDNA sequence electropherogram was manually examined for sequencing artefacts using ClustalW. Alignment demonstrated that ITS2 sequences were highly conserved, with two consistent nucleotide substitutions; a T>C transition at nucleotide (nt) base 479 and a G>T transversion at nt 538. The insertion event at nt 603 was found in all specimens examined (Fig 2). The 17 ITS2 sequences representing each study site are deposited in GenBank accession numbers JN675907- JN675923.

### ITS2 fragment sequence analysis

Sequence alignment revealed a 99.46–99.48% sequence similarity between ITS2 amplicons (n = 152) and the published voucher sequence of *An*. *epiroticus* (GenBank accession number AF469855). Other ITS2 sequences (n = 218) matched either *An*. *sundaicus* sequences from Sarawak, Malaysia (GenBank accession numbers GQ284826, AF369550) or Sumatra, Indonesia (GQ284822). As reported by Dusfour et al. [20, 22], this dataset also revealed that *An*. *epiroticus* ITS2 sequences (BRB-127) and *An*. *sundaicus* differed at several variable sites; a T>C transition at nt 479 and a G>T transversion at nt 538 (Table 1). Variations in *An*. *sundaicus* sequences in this study did not overlap with the variations seen in previous studies or those mentioned above. For example, a sample from western Sumba (SM1-103) possessed a G>T transversion at nt 538 but lacked a base transition at nt 479 (Fig 2). Conversely, the specimens from Enggano Island possessed a T to C transition at nt 479 but lacked a base transversion at nt 538.

**Fig 3:**
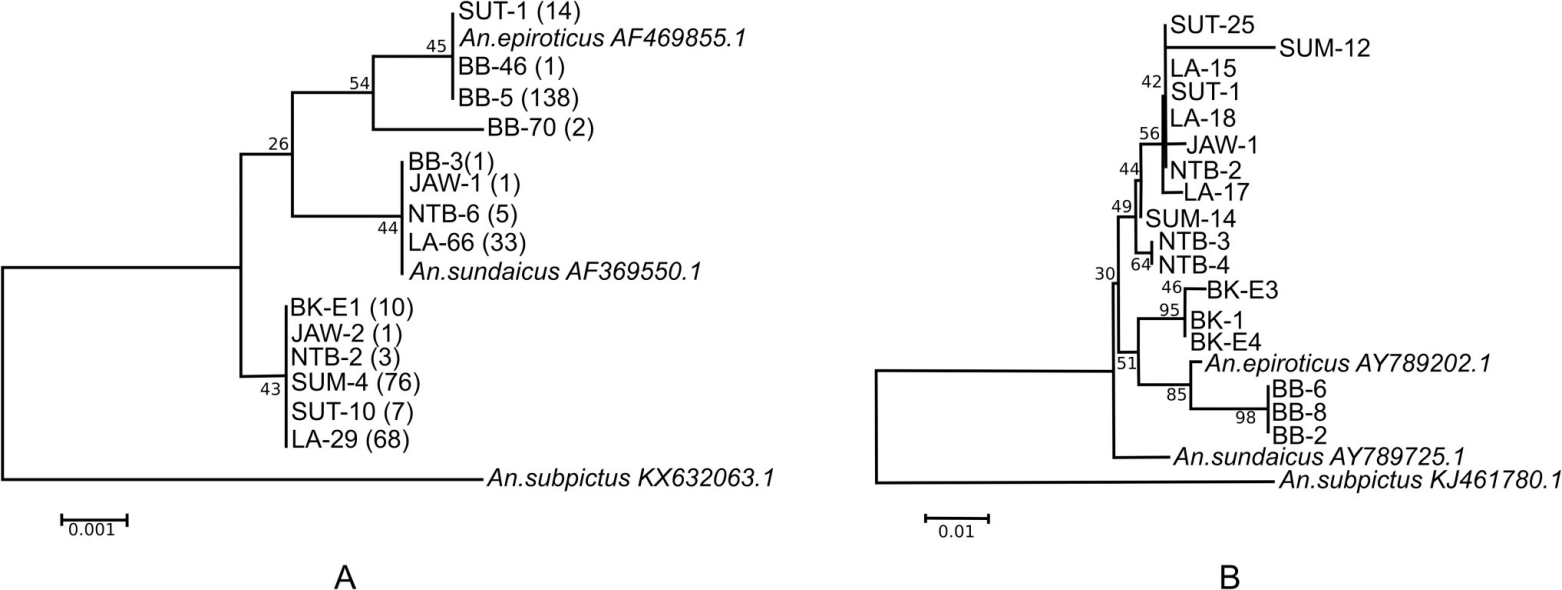
The heteroduplex Y (C/T) at nt 538 of the ITS2 fragment observed in 9 *Anopheles sundaicus* s.l. samples from Bangka-Belitung Province showing evidence of natural species introgression.

Analysis of ITS2 sequences identified polymorphic sites at nt 479 and 538, (Table 1). Based on nucleotides at positions 479 and 538, the *An*. *sundaicus* s.l. specimens possessed four haplotypes: TG, CG, TT, CT. In nine DNA samples from the Bangka-Belitung archipelago, a heteroduplex ITS2 at nt 479 as Y (C/T) and nt 538 as K (T/G/) was detected (Fig 3). *Anopheles epiroticus* appears to exclusively possess the TG haplotypes at these sites [13, 20], and specimens (n = 152) from North Sumatra (Barbaran and Sebajior villages) and Bangka-Belitung each had this distinct haplotype. These mosquitoes, hence, are considered to be *An*. *epiroticus*. The CG and CT haplotypes predominated in all sampled sites, whereas the TT haplotype was only observed on Bangka-Belitung.

### *COI* gene fragment analysis

The *COI* gene fragment (522 bp) was amplified from 107 specimens of *An*. *sundaicus* s.l. Sequence alignment revealed 97 variable sites, of which 32 were parsimony informative. Analysis of nucleotide substitutions revealed no consistent nucleotide position useful to distinguish *An*. *epiroticus* from the other three sibling species. The nucleotide substitution at base 294 where the A to G substitution was present in *An*. *epiroticus* and *An*. *sundaicus* with the lone exception of a single specimen from Bangka-Belitung having the G nucleotide at base 294 (Table 2). No *COI* positions that could be linked to the observed ITS2 sequence differences. Insertion and deletion events were not observed in the sequence alignment analysis.

**Table 2:** Inter-specific variable bases for *COI* and *cytb* genes that distinguish between *Anopheles sundaicus* complex species.

Study Site(Province)	*COI*	*ctyb*					N
	294	12	267	297	469	474	
EPCOI-2/ EPCB-2*	G	C	T	C	T	T	-
SUNCOI-1/SUNCB-1*	A	T	C	T	C	C	-
North Sumatra (EPBRB127)	A	T	T	C	T	C	13
Bangka-Belitung (BB Epi)	G	C	T	C	T	T	26
Bengkulu	G	T	T	C	T	C	10
South Lampung	A	T	T	C	T	C	25
West Java	A	T	T	C	T	C	2
West Nusa Tenggara	A	T	T	C	T	C	8
East Nusa Tenggara	A	T	T	T	C	T	1
	A	T	T	T	T	T	1
	A	T	T	C	T	C	21
Total	-	-	-	-	-	-	107

(*) = [21]

North Sumatra and Bangka Belitung represent *Anopheles epiroticus*, whereas other areas represent *An*. *sundaicus* s.l.

### *Cyt-b* gene fragment analysis

A 485 bp *cytb* gene fragment was amplified from *An*. *sundaicus* s.l. specimens. Alignment of these sequences revealed 128 variable sites, of which 60 were parsimony informative. Alignment of the sequences with published sequences from *An*. *sundaicus* s.s. (GenBank accession numbers AY243796, AY256956, AY299095, AY299098, AY299103) and *An*. *epiroticus* (GenBank accession numbers AY253160, AY256954, JN675907-JN675923) revealed five species-indicative nucleotide variations at bases 12, 267, 297, 469 and 474. However, no variation was unique to a specific species and thus not useful to distinguish between *An*. *epiroticus* and other species complex members (Table 2). With the exception of mosquitoes from western Sumba Island, specimens from all sampled sites were the same haplotype.

### Phylogenetic analysis

**Fig 4:**
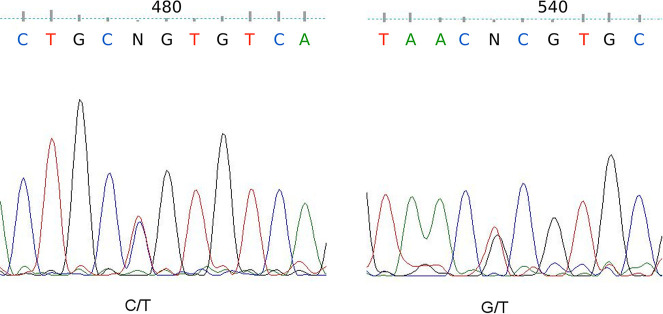
Phylogenetic tree of *An*. *sundaicus* s.l. based on the rDNA ITS2 fragment (Panel A) and concatameric mtDNA *COI* (Panel B). The percentage of replicate trees in which the associated taxa clustered together in the bootstrap test is shown next to the branches. The optimal tree with the sum of branch length = 0.01841176 is depicted. The tree is drawn to scale, with branch lengths in the same units as those of the evolutionary distances used to infer species relationships. Some specimens from North Sumatra (Barbaran and Sebajior) and Bangka-Belitung cluster with *An*. *epiroticus* from Malaysia, Vietnam, and Thailand. Site codes: SUT = North Sumatra; SUM = Sumba, East Nusa Tenggara; BB = Bangka Belitung Archipelago; LA = Lampung; JAW = West Java; NTB = West Nusa Tenggara; and BK = Bengkulu.

All ITS2 and COI sequences obtained from specimens collected from 12 locations were separately aligned with GenBank entries for *An*. *epiroticus* and *An*. *sundaicus* s.s. from Thailand and Malaysia. After removing all ambiguous positions, the sequence datasets used for the final phylogenetic tree reconstruction included ITS2 (n = 17) with sequence length 586 bp and *COI* (n = 20) sequence length 522 bp. The resulting tree with sample names and corresponding GenBank accession numbers is provided (Fig 4). Despite the low bootstrap values for both ITS2 and COI sequences, analysis revealed significantly different clade formation for *An*. *epiroticus* and *An*. *sundaicus* (Fig 4A and 4B respectively). Evolutionary history was inferred using the Neighbor-Joining method [37]. Based on ITS2, specimens from North Sumatra (SUT-11) and Bangka Belitung (BB-46 and BB-5) clustered together with *An*. *epiroticus*. Specimens from West Java, Lampung and other specimens from Bangka Belitung (BB-3) clustered with *An*. *sundaicus* s.s. from Lundu, Sarawak, Malaysia. The vast majority of specimens from Sumba formed a different cluster with specimens from Bengkulu and the remaining specimens from Lampung, North Sumatra, Java and West Nusa Tenggara provinces. *Anopheles epiroticus* may have recently diverged from the *An*. *sundaicus* complex as evidenced by the ITS2 branching (Fig 4A). Based on the *COI* gene, most specimens clustered with *An*. *sundaicus* except for several specimens from Bangka Belitung (BB-2, BB-6 and BB-8) that were closer to *An*. *epiroticus*. Specimens from North Sumatra clustered with *An*. *sundaicus* from Lampung, West Java and West Nusa Tenggara.

## Discussion

Analysis of ITS2 sequences (n = 370) in *An*. *sundaicus* s.l. specimens revealed two previously documented consistent inter-specific variations; a T>C transition at nt 479 and a G>T transversion at nt 538 that distinguish *An*. *epiroticus* from the other members of the Sundaicus Complex. Furthermore, these results corroborate the findings of Linton et al. [21] showing these two nucleotides are suitable to distinguish *An*. *epiroticus* from *An*. *sundaicus*. Linton et al. [21] also reported the absence of a base insertion at ITS2 nt 603 in specimens formerly identified as *An*. *sundaicus* in Vietnam and Thailand. However, this base insertion was found in all specimens from Indonesia, indicating the possibility this mutation event occurred either independent of genetic divergence between *An*. *sundaicus* and *An*. *epiroticus*, or due to periodic recurrent gene flow between landmasses [23].

This study provides the first definitive evidence for the presence and geographical extent of *An*. *epiroticus* in Indonesia, contrary to previous studies that suggested the species exists exclusively in mainland Southeast Asia [21]. Using verified methods [21], *An*. *epiroticus* was documented on the island of Sumatra and the Bangka-Belitung archipelago, islands set in the Java Sea between Sumatra and Kalimantan (Borneo) near the Karimata Strait. These findings also indicate *An*. *epiroticus* occurs in sympatry with other sibling lineages. These observations do not dispute the inferred allopatric speciation of members of the complex on separate Asian landmasses, possibly attributed to repeated geological occurrences of cyclical island and refugium creation. The theory that periodic sea-level changes caused by repeated glaciation events may have created secondary isolation barriers (i.e., vicariance) and genetic bottleneck events during the Pleistocene epoch (2.6 million– 11,700 mya) has been advanced as a biogeographical hypothesis of population history and gene flow in this region [25]. This hypothesis of ecological and allopatric speciation has been challenged based on analyses that indicates species divergence often appears no greater between than within landmasses, thus providing sufficient and recurrent gene flow during recent geologic periods of Earth [23].

The ITS2 nucleotide substitutions used as markers for distinguishing *An*. *epiroticus* from the other members of the *An*. *sundaicus* complex include five haplotypes: TG, CG, TT, CT, and CY. The TG haplotype is associated specifically with *An*. *epiroticus*. At this stage, it is not clear whether the CG, TT, CT, and TY haplotypes correspond with specific forms of *An*. *sundaicus* identified in Indonesia, i.e., cytotypes B and C, *An*. *sundaicus* s.s., and *An*. *sundaicus* E. Interestingly, evidence of heteroduplexes indicate that natural hybridization (introgression) is possible between these sympatric sibling species. However, this evidence does not refute the validity of *An*. *epiroticus* as a distinct taxon as reproductive isolation may breakdown occasionally between sibling species, as seen in other species complexes such as *An*. *gambiae* s.l. where ITS2 sequence-based species differences are maintained and *COI* differences are virtually non-existent [38, 39].

Analysis of *COI* and c*ytb* genes from 97 *An*. *sundaicus* s.l. revealed no consistent species-specific nucleotides useful as molecular markers to distinguish *An*. *epiroticus* from other members of the complex. These findings are not in agreement with Linton et al. (2005) [21] that indicate the A to G transition at base 294 *COI* and nucleotide variations at 12, 267, 297, 469 and 474 *cytb* are useful markers for *An*. *epiroticus*. The use of mtDNA to characterize various organisms, including mosquitoes, is well established [20, 21, 40]. The unique attributes of mtDNA include maternal inheritance, absence of recombination during reproduction (as a single inheritable unit), relatively rapid rates of mutations (evolutionary change), and small size, which makes it a useful marker for examining closely related taxa [41, 42]. However, in this study, variations at nt 27, 122 and 294 of *COI* and at nt 12 of *cytb* could not distinguish *An*. *epiroticus* and the other sibling members. In comparison to the rDNA ITS2 fragment, the mtDNA *COI* and *cytb* genes showed greater variation. This evidence supports the assertion that mtDNA has higher mutation rates than nuclear DNA [43]. Therefore, the *COI* and *cytb* genes may be suitable for distinguishing intra-specific (polytypic) variation within populations.

Of the four cytotypes identified, cytotype A and B are the predominant forms geographically and found in sympatry in various areas of Southeast Asia [20, 21]. Therefore, cross-species gene flow between closely related members of the *An*. *sundaicus* complex is possible and supported by finding naturally occurring heteroduplex ITS2 nucleotides in this study. The heteroduplex (TY haplotype) in nine (~6%) of 151 specimens from Bangka-Belitung indicates that natural introgression can occur between populations of *An*. *sundaicus* haplotypes TT and TC. Naturally occurring hybridization between sibling species, together with other inherent confounders e.g., heteroplasmy, possibility of bi-parental inheritance of mitochondria, and mtDNA introgression [44], may complicate the use of mtDNA-based markers in this complex.

We acknowledge some inherent limitations to the study and analysis. Besides a low number of samples from some localities, having had an opportunity to explore additional molecular markers or approaches would have possibly enhanced the study and conclusions further. However, this was not possible with the material at hand, as this investigation relied solely on specimens collected over many years from different entomological collections and study designs.

The *An*. *sundaicus* complex includes morphologically indistinguishable and epidemiologically important vectors of malaria along coastal areas of South and Southeast Asia [12, 45]. Fortunately, molecular analysis provides a relatively rapid and inexpensive method for accurately identifying many species. This complex includes two formally named species and four forms based on either cytogenetics or rDNA/mtDNA evidence [14–16, 20, 22, 25]. Currently, the complex consists of four cytotypic forms designated A, B, C, and D [16, 19]. *Anopheles sundaicus* species A, present in mainland Southeast Asia was formally named *An*. *epiroticus*, based on DNA sequence differentiation of the entire nuclear ITS2 region and a portion of both mitochondrial *COI* and *ctyb* genes [21]. The use of ITS2 sequences alone may not be robust enough to distinguish between members of the complex, as this gene is highly conserved resulting in a very low bootstrap value upon phylogenetic analyses. Nucleotide differences within the ITS2 gene among the members of the *An*. *sundaicus* complex ranged from 99.4–99.8%—merely 1–3 nt differences. Phylogenetic analysis using ITS2 resulted in three clusters—*An*. *epiroticus*, *An*. *sundaicus* s.s., and the Bengkulu-Sumba cluster. The single or double nucleotide difference seen, combined with sympatric specimens and evidence of possible natural hybridization, results in an unconvincing case to base speciation on this marker alone at these sites. Additional DNA markers with more informative sequences may help clarify these observations. In areas where members of the Sundaicus Complex occur in sympatry, it may be important to accurately distinguish between each species based on possible differing behavioural and biological characteristics influencing capacity to transmit malaria for vector control purposes [2, 46].

Though the major role of *An*. *sundaicus* s.l. in malaria transmission in western Indonesia has long been characterized [10–12], due to its recent discovery, the specific role of *An*. *epiroticus* has not been elucidated. More recently, *An*. *sundaicus* E has been confirmed having a major role in malaria transmission along the south-west coastal region of Sumba island [47]. *Anopheles epiroticus* is a regarded as mostly an incidental or secondary vector in Cambodia, Myanmar, Thailand and Vietnam [48, 49]. The extent to which a vector may contribute to malaria transmission can be site-specific and is likely influenced by prevailing environmental and epidemiological variables such as habitat suitability, seasonality, abundance, blood feeding behaviour (host selection, time of feeding), and vector competence (sporozoite infections) [48, 50]. Compared with other Indonesian vector species, there are limited studies in areas with *An*. *epiroticus* (previously identified as *An*. *sundaicus*) that describe the bionomics and suitable methods of control [51, 52]. Currently, little is known about the specific role of *An*. *epiroticus* in the transmission of malaria in Sumatra and Bangka-Belitung. *Anopheles epiroticus* was not investigated to establish malaria vector status in this study. However, it is possible that mosquitoes morphologically identified earlier as *An*. *sundaicus* s.l. that exhibited relatively high human-biting rates [53, 54] and found infected with malaria sporozoites [53] may actually be *An*. *epiroticus*.

### Conclusions

This study examined the sequence variation of rDNA ITS2 gene fragments and mtDNA *COI* and *cytb* genes of mosquitoes of the *An*. *sundaicus* complex in Indonesia. Previously published species-specific ITS2 nucleotide variations were detected that distinguished *An*. *epiroticus* from other members of the complex. Further studies on the distribution of members of *An*. *sundaicus* s.l. in Indonesia and their potential site-specific roles in malaria transmission requires further investigation. Species-specific identification of members in this complex has practical and possibly operationally relevant significance for targeting vector management programs in the country.
