# Correction: *Anopheles sundaicus* complex and the presence of *Anopheles epiroticus* in Indonesia

**DOI:** 10.1371/journal.pntd.0009345

**Published:** 2021-04-13

**Authors:** Din Syafruddin, Yulia E. Lestari, Dendi H. Permana, Puji B. S. Asih, Brandyce St. Laurent, Siti Zubaidah, Ismail E. Rozi, Sully Kosasih, Shinta, Supratman Sukowati †, Lukman Hakim, Edhi Haryanto, Wibowo Mangunwardoyo, Michael J. Bangs, Neil F. Lobo

In the ITS2 fragment sequence analysis subsection of the Results, there is an error in the fifth sentence of the first paragraph. The correct sentence is: For example, a sample from western Sumba (SM1-108) possessed a G>T transversion at nt 538 but lacked a base transition at nt 479 (Fig 2).

The images for Figs 3 and 4 are incorrectly switched. The image that appears as Fig 3 should be Fig 4, and the image that appears as Fig 4 should be Fig 3. The figure captions appear in the correct order. Please view the images in the correct order here.

**Fig 3:**
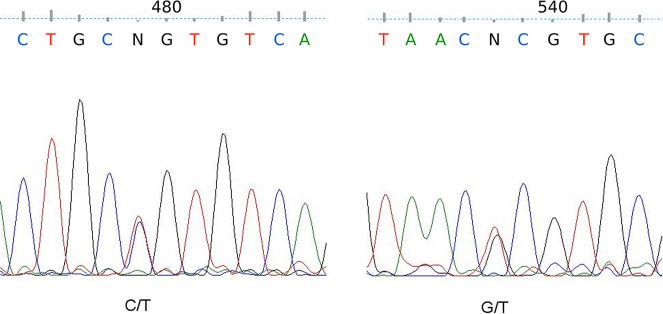
The heteroduplex Y (C/T) at nt 538 of the ITS2 fragment observed in 9 *Anopheles sundaicus* s.l. samples from Bangka-Belitung Province showing evidence of natural species introgression.

**Fig 4:**
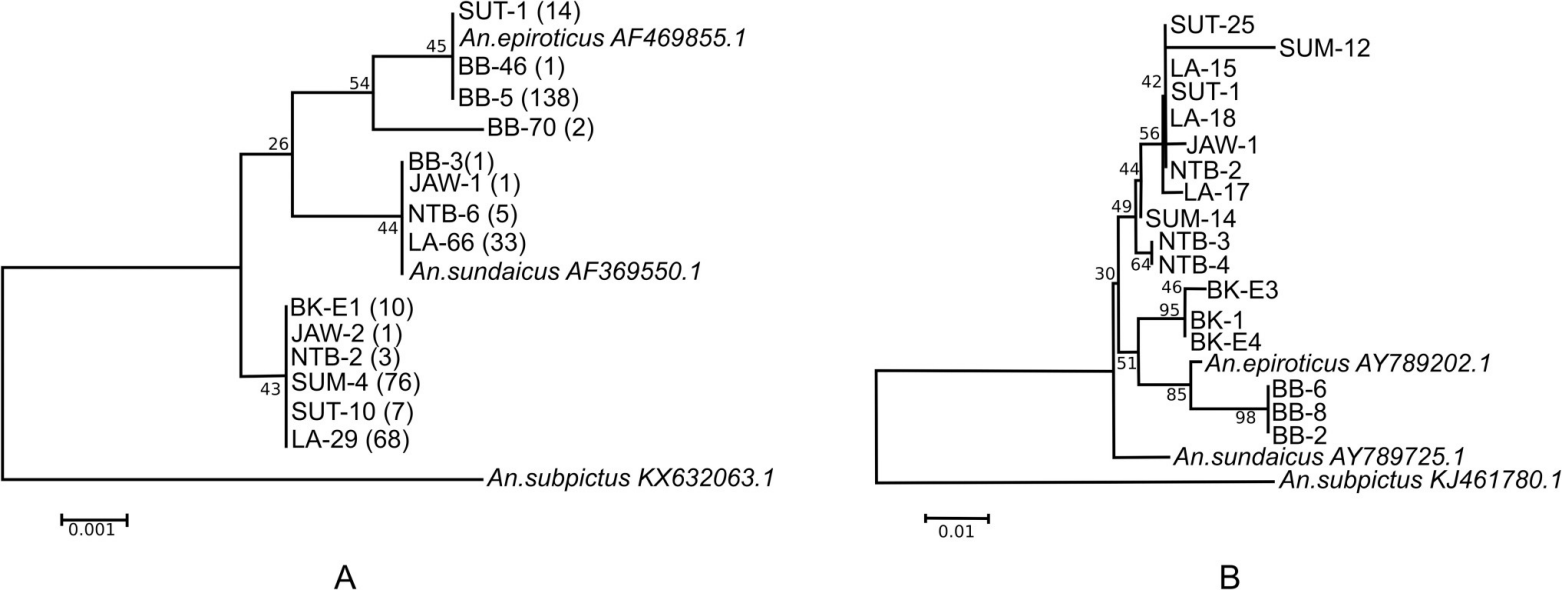
Phylogenetic tree of *An*. *sundaicus* s.l. based on the rDNA ITS2 fragment (Panel A) and concatameric mtDNA *COI* (Panel B). The percentage of replicate trees in which the associated taxa clustered together in the bootstrap test is shown next to the branches. The optimal tree with the sum of branch length = 0.01841176 is depicted. The tree is drawn to scale, with branch lengths in the same units as those of the evolutionary distances used to infer species relationships. Some specimens from North Sumatra (Barbaran and Sebajior) and Bangka-Belitung cluster with *An*. *epiroticus* from Malaysia, Vietnam, and Thailand. Site codes: SUT = North Sumatra; SUM = Sumba, East Nusa Tenggara; BB = Bangka Belitung Archipelago; LA = Lampung; JAW = West Java; NTB = West Nusa Tenggara; and BK = Bengkulu.
